# Responsiveness, construct and criterion validity of the Personal Care-Participation Assessment and Resource Tool (PC-PART)

**DOI:** 10.1186/s12955-015-0322-5

**Published:** 2015-08-12

**Authors:** Susan W. Darzins, Christine Imms, Nora Shields, Nicholas F. Taylor

**Affiliations:** School of Allied Health, Australian Catholic University, 115 Victoria Parade, Melbourne, Fitzroy 3065 Australia; Department of Community & Clinical Allied Health, College of Science, Health and Engineering, La Trobe University, Bundoora, Victoria 3086 Australia; Allied Health Clinical Ressearch Office, Eastern Health, 5 Arnold Street, Box Hill, Victoria 3128 Australia; Northern Health, 1231 Plenty Rd., Bundoora, Victoria 3083 Australia; Murdoch Children’s Research Institute, Royal Children’s Hospital Precinct, 50 Flemington Road, Parkvile, Victoria 3052 Australia; CanChild Centre for Childhood Disability Research, McMaster University, 1280 Main Street West, Hamilton, Ontario Canada

## Abstract

**Background:**

The Personal Care-Participation Assessment and Resource Tool (PC-PART) was designed to measure participation restrictions in activities of daily living required for community life. Rasch analysis has confirmed that the PC-PART contains two unidimensional scales providing interval-level measurement: the *Self Care* and *Domestic Life* scales. This study investigated validity and responsiveness of these PC-PART scales using the COnsensus-based Standards for the selection of health Measurement INstruments (COSMIN) approach.

**Methods:**

Thirteen hypotheses about *Self Care* and *Domestic Life* scale scores were established prior to conducting the analyses. Data from a prospective randomized controlled trial of additional (weekend) inpatient rehabilitation in Melbourne, Australia, were used. The 996 participants had a mean (SD) age of 74 (13) years and were admitted with orthopaedic (n = 581), neurological (n = 203) or other disabling impairments (n = 212). *Self Care* and *Domestic Life* scores were compared to functional independence (FIM), comorbidity (Charlson Comorbidity Index), whether activities of daily living goals were met, and discharge destination.

**Results:**

Low to moderate correlations between FIM and PC-PART scales’ scores supported hypotheses that the PC-PART measures a different construct from functional independence: *Self Care r*_*s*_ -0.52(95 % CI -.46 to -.57) and *Domestic Life r*_*s*_ -0.32(95 % CI -.25 to -.38). The scales had low to moderate discriminative ability for discharge destination, with the area under the curve for *Self Care*, 0.70 (95 % CI 0.62-0.78), and *Domestic Life*, 0.72 (95 % CI 0.64-0.80). The discharge to community living cut-off scores for *Self Care*: 5.50 (sensitivity .83, specificity .53) and *Domestic Life*: 7.50 (sensitivity .75, specificity .60), represented patients having no participation restrictions. Change scores from admission to discharge demonstrated larger effect sizes for the *Self Care* (1.67) and *Domestic Life* (1.50) scales than for the FIM (1.10), supporting hypotheses about responsiveness. Ten of the 13 hypotheses were supported.

**Conclusions:**

This study provided evidence supporting construct validity, criterion validity and responsiveness of the PC-PART *Self Care* and *Domestic Life* scales for inpatient rehabilitation. Clinicians, managers and researchers who wish to measure the patterns and extent of people’s participation restrictions in activities of daily living and the associated burden of care, before and/or after intervention, can be somewhat confident about the PC-PART’s validity and responsiveness for this purpose.

**Trial registration:**

Data used in this research were gathered during a registered randomized controlled trial: Australian and New Zealand Clinical Trials Registry ACTRN12609000973213.

## Background

Rehabilitation aims to improve activity performance and address barriers to patients’ participation in their life situations [1–3]. Rehabilitation services assist patients to adapt to challenges they face in their daily life as a result of their impairments. Participation is a key outcome of rehabilitation programmes [2, 4].

The International Classification of Functioning, Disability and Health (ICF) [5] is a commonly used framework in rehabilitation that informs assessment and measurement of patients’ functioning and health outcomes [6, 7]. The functioning and disability aspect of the ICF framework provides three separate constructs (impairments, activities, participation). However, only two components are described: one for impairments, and one for activities and participation, combined [5]. Researchers have commented on the lack of clarity in the interpretation of, and operationalization of the activity and participation concepts within the ICF framework [3, 7–11]. In particular, there is lack of consensus on interpretation of the definition for, and measurement of, participation-related constructs [7, 8, 10]. It seems accepted that measures eliciting information about an individual’s ability, level of difficulty or level of dependence in performing tasks, without inclusion of the modifying effects of the environment in the instrument’s metric, measure activity limitations [2, 3, 7]. With respect to measurement of participation restrictions, one view is that measures eliciting information about performance of tasks in natural environments and that include influences of the environment on performance in the instrument’s metric, measure participation restrictions [2–4, 12, 13].

The Personal Care-Participation Assessment and Resource Tool (PC-PART) [14–16] was designed to measure the presence or absence of participation restrictions experienced by individuals in self care and domestic activities of daily living (ADL) required for community life. It systematically identifies unmet ADL needs which persist in individuals’ living environments despite their own efforts, use of assistive devices, and supports or assistance from others [14, 16]. The PC-PART provides one conceptual perspective on the measurement of participation restriction in self care and domestic life domains.

The PC-PART differs from commonly used ADL instruments, such as the FIM [17, 18] and the Barthel Index (BI) [19], in a fundamentally important way. The FIM and BI measure patients’ level of dependence in self care and mobility, rating their abilities and their need for assistance or adaptive equipment or both. They can be considered to measure activity limitations [2]. Such instruments are not able to capture what ADL will actually be accomplished. The PC-PART differs in that it measures both the need for assistance or equipment and whether any required assistance is available and is provided in the patients’ living environment. Such information is critical, for example, for discharge planning from inpatient settings [20–23] and for admission decisions in emergency departments [24, 25].

The COnsensus-based Standards for the selection of health Measurement INstruments (COSMIN) is an internationally recognised framework, developed through international consensus of experts in the measurement of health status outcomes [26–28]. The COSMIN checklist provides a framework of criteria for rating the methodological quality of research investigating the reliability, validity, responsiveness and interpretability of health measurement instruments. [27, 29]. It can also guide the development of rigorous methods to investigate measurement properties of health related outcome measures [29].

A systematic review of the measurement properties of the PC-PART using the COSMIN checklist showed that PC-PART items demonstrated good content validity [16, 30]. Other aspects of the instrument’s validity could not be confirmed from the systematic review. Subsequent research has demonstrated that the PC-PART has good inter-rater reliability for group applications but not for individual applications, such as in the clinical setting [31]. Using Rasch methods, a further study generated evidence supporting internal validity of 30 of the original 43 items, when grouped into separate *Self Care* (16 items) and *Domestic Life* (14 items) scales [32]. The objective of this present study was to evaluate the construct validity, criterion validity and responsiveness of the Rasch-derived *Self Care* and *Domestic Life* scales in an adult inpatient rehabilitation setting.

## Methods

### Design

This is an instrument validation study guided by the COSMIN framework, involving secondary analysis of existing data from a prospective randomized controlled trial (RCT). The RCT investigated what effect providing additional Saturday rehabilitation during inpatient rehabilitation had on functional independence, quality of life and length of stay, compared to 5 days per week of rehabilitation [33–35].

### Participants

Participants were the 996 adults enrolled in the trial, conducted in two public hospital multidisciplinary inpatient rehabilitation units in Melbourne, Australia. Participants with orthopaedic (e.g. fractures, elective joint replacements), neurological (e.g. stroke, multiple sclerosis, Parkinson’s disease) or other disabling condition (e.g. cardiac, pulmonary, deconditioning) were included. Patients were not excluded if their primary language was different from English or if they had reduced cognition, but were excluded if they were admitted for ‘Geriatric Evaluation and Management’ (otherwise known as slow stream rehabilitation) or if they were enrolled in another trial. Patients are typically accepted for inpatient rehabilitation if assessed as being able to participate actively in rehabilitation with the expectation they will improve sufficiently to return to community living [33]. Ethics approval for this study, involving secondary analysis of the RCT data, was received from University and Health Service Human Research Ethics Committees. Participants gave written informed consent to take part in the RCT.

### Measures

Data from all measures used in the RCT were available for this study and these are detailed elsewhere [33]. Hypotheses for this present study were generated prior to all analyses with knowledge of the available measures used for the RCT. Only data from measures relevant to the hypotheses for this present study were used.

The **PC-PART** was administered to gather data for the RCT at admission (baseline) and again at discharge from inpatient rehabilitation. It was administered by occupational therapists using a combination of patient interview, key informant interview and task observation (see Appendix A: Table 8 for item examples). Prior to commencement of data collection the occupational therapist assessors were provided with standardized education in the use of the PC-PART. This consisted of a one-hour training session. In addition, the occupational therapists were provided the PC-PART manual [14] and a recorded audiovidual presentation [15].

The PC-PART assessment was administered to identify participants’ existing participation restrictions in ADL in their discharge living environments. Items were scored as either *OK by self* (patients will manage the activity alone with or without aids in their living environments), *OK with help* (patients will manage the activity with help from others, and this help is available and provided in their living environments), or *Not OK* (patients will not manage the activity in their living environments despite their own efforts, use of aids and help from available support from others). Both *OK by self* and *OK with help* were scored 0 (*no* participation restriction present), and *Not OK* was scored 1 (participation restriction *was* present). Each *Not OK* represented an ADL participation restriction. These item response categories were shown to be valid using Rasch analysis [32]. When used clinically, the raw score for each scale is the total number of *Not OK* scores observed for an individual patient, with a range of possible scores of 0–16 (*Self Care*) and 0–14 (*Domestic Life*). Rasch-derived conversion scores for each scale use a 0–100 scale, where 0 reflects no ADL participation restriction and 100 represents complete ADL participation restriction. *Self Care* and *Domestic Life* scale total scores cannot be combined to form an overall PC-PART score [32].

Rasch-derived scores for the 16 *Self Care* scale items and 14 *Domestic Life* scale items were used for all analyses in this present study [32]. To aid clinical interpretation where relevant, Rasch-derived scores were related back to corresponding total raw *Self Care* and *Domestic Life* scores using a conversion table [32].

The **FIM** [36] consists of 18 items from motor (13 items) and cognitive (5 items) domains. Each item is rated on a 7-point scale, where 1 represents complete dependence and the need for total assistance and 7 represents complete independence. Scores range from 18 (complete dependence on all items) to 126 (complete independence on all items) [37]. Items cover activities such as eating, grooming, bathing, dressing, toileting, sphincter control, , transfers, locomotion, communication and social cognition. There is evidence from studies conducted in the past two decades across several countries and different patient populations, supporting reliability, validity and responsiveness of the FIM as a measure of disability for patients receiving rehabilitation [38]. Thus, the FIM was viewed as a suitable comparison instrument for the PC-PART. It is a measure of activity limitations according to ICF concepts and terminology [5, 39]. It has sufficient similarity in the content of its domains to the PC-PART, to generate hypotheses reflecting expected convergence and divergence between their scores at admission and discharge from inpatient rehabilitation. The FIM was administered as part of routine care by FIM trained assessors, including physiotherapists and occupational therapists. It was scored during multidisciplinary team meetings at admission (baseline) and at discharge from inpatient rehabilitation. At both points, the FIM was completed on a separate occasion to the PC-PART.

The **Charlson Comorbidity Index** [40] was selected as the best available measure used in the RCT to enable testing of hypotheses about the PC-PART’s scores related to the level of patients’ co-morbidity. The sum of the index score, adjusted for age, is an indicator of disease burden and an estimator of mortality [40]. It provided a mechanism to quantify the severity of a patient’s overall state of ill-health, given the number and seriousness of health conditions experienced. The index has been widely used and validated in population studies [41], but it is recognized that some conditions (e.g. rheumatological disease) are less accurately coded [42]. The score was calculated at admission.

ADL discharge goals were established by the patients and treating occupational therapists at admission. This information was gathered for the RCT but not with the structured approach of goal attainment scaling. **Achievement of ADL goals** was measured and recorded at discharge from inpatient rehabilitation by the treating occupational therapist as being either met/unmet. Partially met goals were categorized as unmet.

Patients’ **discharge destinations**, that is, patients’ living situations immediately following discharge from inpatient rehabilitation were categorised as *home* (usual place of residence), *low-level residential care*, *high-level residential care*, *acute hospital transfer*, or *transitional care*. The transitional care program involved continued inpatient care for either lower intensity rehabilitation activities or to await placement in residential care facilities.

### Analysis

#### COSMIN checklist

The COSMIN checklist provided criteria for evaluating construct validity, criterion validity and responsiveness. In this study all design criteria were addressed [29, 43].

#### Construct validity

COSMIN stipulates that construct validity is the degree to which the scores of health related outcome instruments are consistent with hypotheses formulated prior to data analysis, based on the assumption that the instrument measures the construct of interest [27]. In accordance with COSMIN’s recommendations, construct validity was evaluated by five hypotheses of expected mean score differences between impairment groups, and expected correlations between PC-PART scores and FIM and Charlson Comorbidity Index scores [29]. The hypotheses and statistical test criteria used are presented in Table 1.

**Table 1:** Methods: Hypotheses, criteria and rationale used to test construct validity, criterion validity and responsiveness

Construct tested	Hypothesis number	Hypotheses about *Self Care*	Hypotheses about *Domestic Life*	Rationale	Test Criteria used
Construct validity	1	At admission, there will be a large negative correlation between *Self Care* scores and FIM total scores, for the whole sample.	At admission there will be a moderate negative correlation between *Domestic Life* scores and FIM total scores for the whole sample.	Higher correlations expected between *Self Care* scale and FIM than between *Domestic Life* and FIM. *Self Care* scale contains more items with content directly related to the FIM than *Domestic Life* scale and appears to measure same construct at high levels of functioning (i.e. ‘OK by self’ on the PC-PART and scores of 6–7 on the FIM).	Magnitude of correlation coefficient (*r*_*s*_)^a^: *r*_*s*_ ≥ .5 = large, *r*_*s*_ .3 to .49 = moderate, *r*_*s*_ .1 to .29 = small [44].
	2	At admission, there will be a large negative correlation between *Self Care* scores and FIM total scores, irrespective of sex, age and major impairment groups	At admission, there will be a a moderate negative correlation between *Domestic Life* scores and FIM total scores, irrespective of sex, age and major impairment groups.		
	3	There will be a moderate positive correlation between admission *Self Care* and Charlson Comorbidity Index scores.	There will be a moderate positive correlation between admission *Domestic Life* scores and Charlson Comorbidity Index scores.	Patients with high co-morbidity expected to have more ADL activity limitations and more support needs than patients with low comorbidity. More support needs expected to be more difficult to satisfy, resulting in higher levels of ADL participation restriction than for those with low comorbidity.	
	4	On admission, there will be no observed differences in *Self Care* participation restriction scores between patient impairment groups.	On admission, there will be no observed differences in *Domestic Life* participation restriction scores between patient impairment groups.	Differences in scores between impairment groups not expected because PC-PART measurement records interactions between persons, tasks and environment. Scores not based on patients’ impairments or diagnoses.	Admission *Self Care* and *Domestic Life* Mean ± 95 % CI scores for each impairment group.
	5	*Self Care* mean discharge scores will be lower for patients who attained their ADL goals than for patients who did not attain their ADL goals by at least one participation restriction on the *Self Care* scale.	*Domestic Life* scale mean discharge scores will be lower for patients who achieved their ADL goals than for patients who did not achieve their ADL goals by at least one participation restriction on the *Domestic Life* scale.	Patients’ inpatient rehabilitation ADL goals focused on optimising independence in self-care and domestic life activities of daily living and arranging zappropriate supports to enable discharge to the community. Achievement of ADL goals therefore expected to correspond to low *Self Care* and *Domestic Life* participation restriction (unmet needs) scores.	Mean difference in 1 Rasch-derived participation restriction scores: *Self Care* = 6.3 *Domestic Life* = 6.9. Differences assessed using 95 % CI mean estimates.
Criterion Validity	6	*Self Care* scales will discriminate between those patients discharged to ‘home or residential care’ versus patients discharged to ‘acute hospital or transitional care.’	*Domestic Life* scales will discriminate between those patients discharged to ‘home or residential care’ versus patients discharged to ‘acute hospital or transitional care’	‘Gold standard’ of ‘discharge destination’is the criterion for estimating the probability that *Self Care* and *Domestic Life* scale scores are an accurate reflection of discharge destination. Theoretical expectation is thatpatients discharged to community living situation (home, low- or high-level residential care) will have resolved ADL participation restrictions. Patients discharged to acute hospital or transitional care are likely to have unresolved ADL participation restrictions.	Area under the curve (AUC) range is 1.0 (perfect discrimination) to .5 (no discrimination): >.9 = high; .7 to .9 = moderate; >.5 to .69 = low; .5 = none [45]
	7	Patients discharged home or to residential care will have mean scores on the discharge *Self Care* scale reflecting less than three ADL participation restrictions.	Patients discharged home or to residential care will have mean scores on the discharge *Domestic Life* scale reflecting less than three ADL participation restrictions.	Gold standard’ is ‘discharge destination’. Predicted cut-off scores reflecting three participation restrictions was a conservative, low estimate.	Rasch derived scores representing 3 ADL participation restrictions: *Self Care* = 25 *Domestic Life* = 33
	8	Patients discharged to acute hospital care or transitional care will have mean scores on the discharge *Self Care* scale reflecting three or more ADL participation restrictions.	Patients discharged to acute hospital care or transitional care will have mean scores on the discharge *Domestic Life* scale reflecting three or more ADL participation restrictions.	Gold standard’ is ‘discharge destination’. Predicted cut-off scores reflecting three participation restrictions was a conservative, low estimate.	
Responsive-ness	9	There will be a low to moderate negative correlation between change scores on the *Self Care* scale and the FIM change score across the whole sample.	There will be a low to moderate negative correlation between change scores on the *Domestic Life* scale and the FIM change score across the whole sample.	*Self Care* and *Domestic Life* scores expected to show greater reduction in scores than relative increase in FIM scores because patients’ ADL participation restrictions expected to be resolved at discharge to enable return to community living, reflecting PC-PART scale scores at/close to zero at discharge. Relatively small improvements in FIM scores between admission and discharge can be observed for patients discharged to community, provided adequate supports are provided.	Magnitude of correlation coefficient (*r*_*s*_)^a^: *r*_*s*_ ≥ .5 = large, *r*_*s*_ .3 to .49 = moderate, *r*_*s*_ .1 to .29 = small [44].
	10	There will be a low to moderate negative correlation between change scores on the *Self Care* scale and the FIM change score irrespective of sex, age and major impairment groups.	There will be a low to moderate negative correlation between change scores on the *Domestic Life* scale and the FIM change score irrespective of sex, age and major impairment groups.		
	11	The effect size observed on the *Self Care* and the FIM between admission and discharge will each be large, but the effect size observed on the FIM will be lower than that of the *Self care* scale.	The effect size observed on the *Domestic Life* scale and the FIM between admission and discharge will each be large, but the effect size observed on the FIM will be lower than that of the *Domestic Life* scale.		Effect size (ES) = (discharge mean – admission mean)/SD admission mean. Effect sizes: .2 = small; .5 = medium & .8 = large [44]
	12	For patients discharged to ‘home or residential care’, there will be a large effect size on the *Self Care* scale.	For patients discharged to ‘home or residential care’, there will be a large effect size on the *Domestic Life* scale.		
	13	The effect size on the *Self Care* scale for those discharged to ‘acute hospital or transitional care’ will be small to medium.	The effect size on the *Domestic Life* scale for those discharged to ‘acute hospital or transitional care’ will be small to medium.		

^a^Spearman correlation used to accommodate ordinal FIM data

#### Criterion Validity

COSMIN stipulates that criterion validity is the degree to which the scores of a health related patient reported outcomes instrument are an adequate reflection of a suitable *gold standard* [27]. In this case, the objectively observable dichotomous *gold standard* outcome was discharge destination (community living at home or in residential care versus inpatient acute or transitional care), reflecting an overall aim of rehabilitation to prepare patients for community living. Criterion validity was tested using three hypotheses, in accordance with COSMIN recommendedations. The hypotheses are presented in Table 1.

Receiver-Operator Characteristic curve data were used to estimate cut-off scores at discharge for the *Self Care* and *Domestic Life* scales that may discriminate patients discharged home or to residential care from those transferred to acute hospital or transitional care. Consideration was given to balancing sensitivity and specificity of the scales’ scores.

#### Responsiveness

COSMIN stipulates that responsiveness is the ability of an instrument to detect change over time when change has occurred [27]. In accordance with COSMIN’s recommendations, responsiveness was evaluated with five hypotheses about the relationship between change scores on the PC-PART and FIM and predicted magnitudes of effect sizes of each measure between admission and discharge (see Table 1).

#### Data analysis

Data were analyzed using IBM SPSS Statistics (Version 21.0.0) software. Missing study data were removed from analyses using pairwise methods in all analyses. According to the COSMIN rating scale [46], a sample size for testing measurement properties of n ≥ 100, is considered *excellent*; from n = 50-99 is *good*; from n = 30-49 is *fair*; and n < 30 is *poor*. It was expected that sample sizes, per analysis, using the RCT data (n = 996) would be *excellent* for evaluating construct validity, criterion validity and responsiveness of the Rasch-derived *Self Care* and *Domestic Life* scales.

## Results

The 996 participants had a mean (SD) age of 74 (13) years, and 631 (63 %) were women (see Table 2). There were 581 (58 %) participants admitted with an orthopaedic diagnosis, 203 (20 %) with a neurological diagnosis and 212 (21 %) with other disabling impairments. Mean (SD) length of stay in the rehabilitation unit was 21 (16) nights. Most participants (93 %) were living at home prior to their acute hospital admission. Of the 7 % of participants not living at home prior to admission, 2 % (n = 27) lived in low-level residential care (LLC), 2 % (n = 23) lived in ‘other’ accommodation, and 2 % (n = 19) had missing data for this variable. Participants from LLC or ‘other’ accommodation (n = 50) showed average (median) improvement of 18 points on the FIM from admission to discharge.

**Table 2:** Participant characteristics and study data

Characteristic	Men	Women	All
Gender: n (%)	365 (37)	631 (63)	996 (100)
Age in years: mean (SD),	73 (13),	75 (13),	74 (13),
min, max	33, 98	22, 102	22, 102
Age group: n (%)			
≤59 years	57 (16)	78 (12)	135 (14)
60 to 79 years	180 (50)	292 (46)	472 (47)
≥80 years	128 (35)	261 (41)	389 (39)
Living at home prior to admission:			
n (%), missing	341 (93), 12	586 (93), 7	927 (93), 19
Length of stay^a^: mean (SD),	22 (17),	21 (15),	21 (16),
n, min, max, missing	359, 3, 124, 6	626, 3, 144, 5	985, 3, 144, 11
Impairment category: n (%)			
Stroke	88 (24)	72 (11)	160 (16)
Other neurological	20 (6)	23 (4)	43 (4)
Orthopaedic	171 (47)	410 (65)	581 (58)
Pain syndromes	12 (3)	31 (5)	43 (4)
Cardiac/Pulmonary	24 (7)	24 (4)	48 (5)
Other disabling impairments	50 (14)	71 (11)	121 (12)
Charlson Comorbidity Index: mode, median	0,1	0,0	0,1
Quartiles (25th ,50th ,75th)	0,1,2	0,0,1	0,1,2
min, max	0,9	0,9	0,9
PC-PART *Self Care* scores^b^:			
Admission: mean score (SD)	41.6 (24.4)	42.3 (21.0)	42.0 (22.3)
min, max, missing	0, 100, 11	0, 100, 27	0, 100, 38
Discharge: mean score (SD)	4.6 (12.1)	3.5 (11.1)	3.9 (11.5)
min, max, missing	0, 100, 42	0, 100, 58	0, 100, 100
PC-PART *Domestic Life* scores^b^:			
Admission: mean score (SD)	38.1 (22.5)	38.7 (19.0)	38.5 (20.4)
min, max, missing	0, 100, 11	0, 100, 28	0, 100, 39
Discharge: mean score (SD)	9.3 (17.1)	6.8 (14.3)	7.7 (15.4)
min, max, missing	0, 100, 36	0, 100 57	0, 100, 93
FIM total scores^c^:			
Admission: median,	86,	87,	87,
mean score (SD)	81.9 (22.2)	85.1 (17.4)	83.9 (19.3)
min, max, missing	18, 124, 0	23, 122, 1	18, 124, 1
Discharge: median,	110,	112,	111,
mean score (SD)	102.8 (21.1)	106.6 (16.0)	105.2, (18.1)
min, max, missing	18, 125, 6	18, 126, 3	18, 126, 9
Were ADL goals met at discharge?			
Yes: n (%)	241 (66)	482 (76)	723 (73)
No: n (%)	100 (27)	116 (18)	216 (22)
Missing: n (%)	24 (7)	33 (5)	58 (6)
Discharge destination			
Home: n (%)	289 (79)	505 (80)	794 (80)
Low level residential care: n (%)	10 (3)	33 (5)	43 (4)
High level residential care: n (%)	16 (4)	20 (3)	36 (4)
Acute hospital transfer: n (%)	10 (3)	7 (1)	17 (2)
Transitional Care Prog. and ‘other’: n (%)	25 (7)	44 (7)	69 (7)
Missing: n (%)	15 (4)	22 (4)	38 (4)

^a^Number of nights in inpatient rehabilitation

^b^Interval level scale 0 to 100, where 0 reflects no ADL participation restriction, 100 reflects highest level of ADL participation restriction

^c^Ordinal scale from 18 to 126, where 18 reflects total dependence, 126 reflects total independence

Approximately 10 % of discharge PC-PART data for both *Self Care* and *Domestic Life* scales were missing (see Table 2). There were a number of these patients for whom most discharge PC-PART individual item data were available, but for whom *Self Care* scores (n = 64) and *Domestic Life* scores (n = 59) could not be calculated because there was between one and three missing values for individual items in the scale. To use a Rasch-derived scale and its associated conversion table, all items in the scale need to be completed to produce a valid score. There were 34 patients (3 % of the sample) with no discharge PC-PART data. Patients with no discharge PC-PART data (n = 34) had similar mean age (74 yrs, SD = 15, 95 % CI 67–81), length of stay (20 nights, SD = 21, 95 % CI 16–41), admission *Self Care* scale scores (Mean = 48.0, SD = 25.1, 95 % CI 40.7-63.4) and *Domestic Life* scale scores (Mean = 38.4, SD = 19.5, 95 % CI 29.3-50.2) compared to the rest of the sample. However, their admission FIM scores (median = 72) and discharge FIM scores (median = 71) were lower compared to the rest of the sample. A higher proportion of patients with no discharge PC-PART data were discharged to acute care (33 %), compared to 2 % for the whole sample.

### Construct validity

#### Hypothesis 1

Table 3 shows that at admission, the large negative correlation between *Self Care* scores and FIM total scores, *r*_*s*_ = −.52 (95 % CI -.46 to-.57), and a moderate negative correlation between *Domestic Life* scores and FIM total scores, *r*_*s*_ = −.32 (95 % CI -.25 to-.38), supported the hypothesis about the magnitude and direction of these correlations. However, 95 % confidence intervals of the estimates included lower correlations than expected.

**Table 3:** Hypotheses 1 and 2 (construct validity): correlations between PC-PART scales and FIM at admission to inpatient rehabilitation

	Spearman correlation: *r*_*s*_ (95 % CI)^a^			Hypothesis supported? *Self Care*: *r*_*s*_ ≥ .5?^b^
				*Domestic Life*: *r*_*s*_ .30 to .49?^b^
**Whole sample**	**n = 956**			
*Self Care* and FIM	.52(.46,.57)			Yes^c^
*Domestic Life* and FIM	.32(.25,.38)			Yes^c^
**by Sex**	**Women n = 602**	**Men n = 354**		
*Self Care* and FIM	.51(.44,.57)	.53(.44,.61)		Yes^c^
*Domestic Life* and FIM	.32(.24,.39)	.32(.22,.42)		Yes^c^
**by Age**	**≤59 yrs**	**60 to 79 yrs**	**≥80 yrs**	
	n = 127	n = 454	n = 375	
*Self Care* and FIM	.52(.35,.65)	.51(.42,.59)	.44(.34,.53)	Yes: ≤59yrs^c^ & 60 to 79 yrs^c^
				No: ≥80yrs^e^
*Domestic Life* and FIM	.37(.21,.53)	.30(.21,.39)	.28(.18,.37)	Yes: ≤59yrs^c, d^ & 60 to 79 yrs^c^
				No: ≥80yrs^e^.
**by Impairment**	**Orthopaedic** **n = 561**	**Neurological** **n = 194**	**Other Impairments ** **n = 201**	
*Self Care* and FIM	.41(.33,.48)	.70(.59,.79)	.48(.35,.58)	Yes: Neurological.
				No: Orthopaedic.
				No: Other Impairment^e^
*Domestic Life* and FIM	.27(.18,.34)	.40(.26,.52)	.28(.14,.41)	Yes: Neurological^c,d^,
				No: Orthopaedic^e^ & Other Impairment^e^

^a^Absolute magnitude of the negative correlation values are represented

^b^Using Cohen’s definition[44]: *r*_*s*_ = .10 to .29 (small); *r*_*s*_ = .30 to .49 (medium); *r*_*s*_ = .50 to 1.0 (large)

^c^Lower bound 95 % confidence interval suggests true value potentially lies below the range specified

^d^Upper bound 95 % confidence interval suggests true value potentially lies above the range specified

^e^Upper bound 95 % confidence interval suggests true value potentially lies within the range specified

#### Hypothesis 2

Correlations by sex, age, and impairment between PC-PART scales and FIM generated 16 correlation values. The magnitude of 10 correlation values were as hypothesized, but six correlation values were lower than expected for both PC-PART scales (participants aged ≥80 years; those with orthopaedic or other disabling impairments) (see Table 3). Fifteen of the 16 lower bound 95 % confidence interval estimates were lower than predicted. Two upper bound 95 % confidence interval estimates for *Domestic Life* and FIM were higher than expected (participants aged ≤59 years; those with neurological impairment).

#### Hypothesis 3

There was a negligible (< .1) to small (.10 to.29) positive correlation between admission *Self Care, r*_*s*_ = .10 (95 % CI .04-.16), and *Domestic Life*, *r*_*s*_ = .04 (95 % CI .02-.10) scores and Charlson Comorbidity Index scores, suggesting a negligible relationship between the PC-PART scales and degree of comorbidity. This result did not support the hypothesis of a moderate positive correlation between the variables. Post hoc analysis showed that 75 % of participants’ comorbidity scores were ≤2 and 50 % of scores were ≤1, showing relatively low variation in scores across the sample.

#### Hypothesis 4

**Fig. 1:**
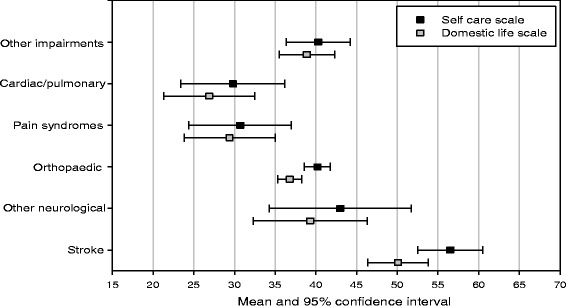
Hypothesis 4 (construct validity): PC-PART *Self Care* and *Domestic Life* scores at admission for impairment groups, displaying mean and 95 % confidence interval for each group. (see separate file)

The hypothesis of no difference in *Self Care* and *Domestic Life* scale mean scores across impairment groups was not supported. The mean scores and 95 % confidence intervals from the group of patients with stroke [*Self Care* 56.5(95 % CI 52.5-60.5); *Domestic Life* 50.1 (95 % CI 46.4-53.8)] demonstrated higher admission scores on both PC-PART scales than patients in other impairment groups, with the closest group being patients with other neurological conditions [*Self Care* 43.0 (95 % CI 34.3-51.7); *Domestic Life* 39.3 (95 % CI 32.2-46.3)] (see Fig. 1).

#### Hypothesis 5

The mean difference in PC-PART scores between patients who attained their ADL goals and patients who did not, was 9.3 (95 % CI 6.6-12.1) for *Self Care* and 12.2 (95 % CI 9.0-15.4) for *Domestic Life* ( see Table 4). As hypothesized, these values represented a clinically relevant difference in raw scores of at least one ADL participation restriction between groups on each scale.

**Table 4:** Hypothesis 5 (construct validity): PC-PART scores and 95 % CIs at discharge for variable ‘ADL goal met?’

PC-PART scale:	ADL goal met?	No (n = 193)	Difference between Means (95 % CIs)	Is difference > 1 ADL participation restriction?
	Yes (n = 679)			
	Mean scale score (95 % CI)	Mean scale score (95 % CI)		*Self Care* ≥ 6.3?^a^ *Domestic Life* ≥ 6.9? ^a^
*Self Care*	1.7 (1.3-2.1)	11.0 (8.3-13.8)	9.3 (6.6-12.1)	Yes
*Domestic Life*	4.6 (3.8-5.3)	16.8 (13.7-19.9)	12.2 (9.0-15.4)	Yes

^a^Value represents the mean difference between any two participation restriction scores on the 0 to 100 Rasch-derived conversion scale

### Criterion validity

#### Hypothesis 6

**Fig. 2:**
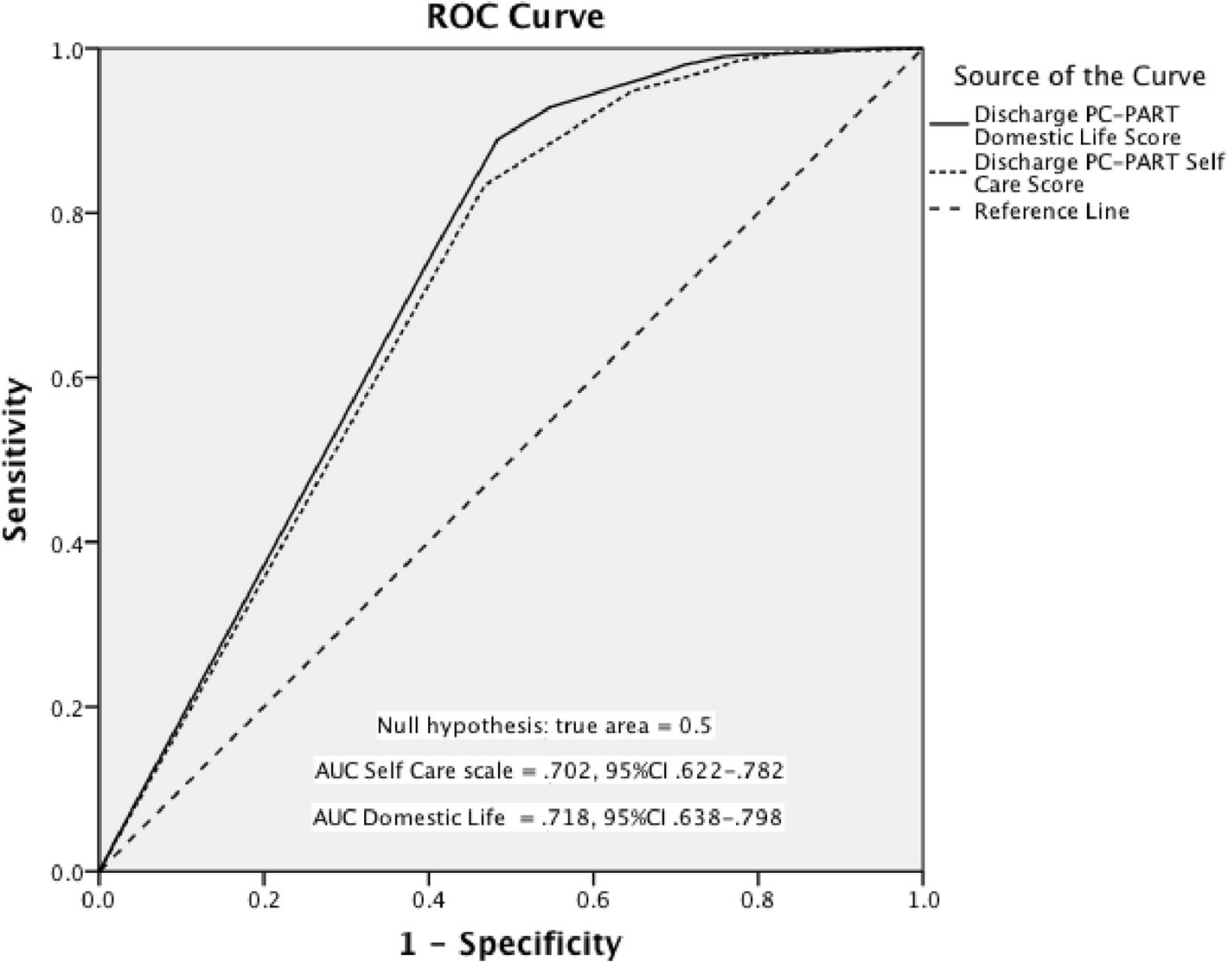
Criterion validity: Area under the ROC curve (AUC) for the discharge PC-PART *Self Care* and *Domestic Life* scores, discriminating between patients discharged to home or residential care (community living) and patients discharged from inpatient rehabilitation to continued inpatient care. (see separate file)

Both *Self Care* and *Domestic Life* scale scores demonstrated low to moderate probability of correctly differentiating between patients discharged home or residential care (n = 815) versus patients discharged to acute hospital or transitional care (n = 86). The estimated area under the curve for the *Domestic Life* scale was .72 (95 % CI: .64-.80) and for the *Self Care* scale, was .70 (95 % CI: .62-.78). This result was modest, but supported the hypothesis of an area under the curve greater than .50, representing discriminative ability greater than chance (see Fig. 2).

#### Hypothesis 7

The hypothesis that those discharged home or to residential care would have *Self Care* and *Domestic Life* discharge scores representing less than three ADL participation restrictions, was supported (see Table 5). Those discharged to community living (home, low level-, high level residential care) had discharge mean *Self Care* scores of 2.7 (95 % CI 2.2-3.3), and *Domestic Life* scores of 6.2 (95 % CI 5.3-7.0), representing raw scores of no ADL participation restrictions on each scale.

**Table 5:** Hypotheses 7 and 8 (criterion validity): PC-PART Rasch-derived scores, raw scores and 95 % CIs at discharge, by discharge destination

PC-PART scale:	Discharge to:	Rasch score:	Discharge to:	Rasch score:
	Home, LLC, HLC (n = 815)	Self Care <25?	Acute care, TCP (n = 86)	Self Care ≥25?
	Mean score (95 % CI)	Domestic Life < 33?	Mean score (95 % CI)	Domestic Life ≥ 33?
		Raw score <3 both scales?		Raw score ≥3 both scales?
*Self Care*:				
Rasch conversion scores	2.7 (2.2-3.3)	Yes	18.4 (11.5-25.3)	No^a^
Equivalent raw scores	0 (0–0)	Yes	1 (1–3)	No^a^
*Domestic Life:*				
Rasch conversion scores	6.2 (5.3-7.0)	Yes	27.5 (20.1-34.8)	No^a^
Equivalent raw scores	0 (0–0)	Yes	3 (1–3)	Yes^b^

Low-level residential care (LLC); High-level residential care (HLC); Transitional Care Program (TCP) ^a^ Upper bound 95 % confidence interval suggests true value potentially lies in the range specified

^b^Lower bound 95 % confidence interval suggests true value potentially lies below the range specified

#### Hypothesis 8

The hypothesis that those discharged to acute or transitional care would have *Self Care* and *Domestic Life* discharge mean scores representing three or more ADL participation restrictions, was partly supported. The 95 % confidence intervals included scores representing three ADL participation restrictions, but also included the possibility of two or one ADL participation restrictions. Those discharged to acute hospital or transitional care had discharge mean *Self Care* scores of 18.4 (95 % CI 11.5-25.3), and *Domestic Life* scores of 27.5 (95 % CI 20.1-34.8), representing raw scores of one to three ADL participation restrictions on each scale (see Table 5). Post-hoc analysis for this combined group showed that 13 of the 17 patients discharged to acute care had no discharge PC-PART data. The other four patients discharged to acute care for whom discharge PC-PART data were available, had at least 14 *Self Care* participation restrictions and 12 *Domestic Life* participation restrictions at discharge. Of the 69 patients discharged to transitional care, 30 (44 %) had no *Self Care* participation restrictions and 26 (38 %) had no *Domestic Life* participation restrictions.

### Cut-off scores

Table 6 shows potential cut-off scores for each scale at several levels of sensitivity to correctly identify patients discharged to home or to residential care. Corresponding levels of specificity for scores to correctly identify patients discharged to acute hospital or transitional care are provided. Cut-off scores of zero on both PC-PART scales reflected optimal sensitivity but specificity values were relatively low : *Self Care* 5.50 (Sensitivity, .83, Specificity, .53), and *Domestic Life* 7.50 (Sensitivity, .75, Specificity, .60).

**Table 6:** Criterion validity: Discharge *Self Care* and *Domestic Life* scale ROC cut-off scores and their corresponding sensitivity/specificity in identifying discharge destination

PC-PART scale	Positive if ≤ to^a^:	Raw scores represented	Sensitivity	1-Specificity	Specificity
			(true + ve)	(false + ve)	(true -ve)
Discharge *Self Care* score	−1.00	<0	.00	.00	1
	5.50	0	.83	.47	.53
	15.00	1	.95	.64	.46
	22.00	2	.97	.74	.26
Discharge *Domestic Life* score	−1.00	<0	.00	.00	1
	7.50	0	.75	.40	.60
	20.50	1	.90	.48	.52
	29.50	2	.93	.55	.45
	35.50	3	.96	.65	.35

Potential cut-off scores to optimise sensitivity and specificity of PC-PART scales for identifying patients with ADL participation restrictions who should remain as inpatients and those who may appropriately be discharged to a specified community living situation

^a^Positive state is discharge to home or residential care. The smallest cutoff value is the minimum observed test value minus 1, and the largest cutoff value is the maximum observed test value plus 1. All the other cutoff values are the averages of two consecutively ordered observed test values

### Responsiveness

#### Hypothesis 9

As hypothesized, there was a low to moderate negative correlation (*r*_*s*_ ≤ −.49) across the sample, between FIM change scores and *Self Care* change scores, *r*_*s*_ = −.40 (95 % CI -.34 to -.45), and *Domestic Life* change scores, *r*_*s*_ = −.22 (95 % CI -.16 to-.30) (see Table 7).

**Table 7:** Hypotheses 9 and 10 (responsiveness): correlations between PC-PART scales’ change scores and FIM change scores, between admission and discharge

	Spearman correlations			Hypothesis supported?
	*r*_*s*_ (95%CI)^a^			*r*_*s*_ ≤ .49?^b^
**Whole sample**	**n = 891**			
*Self Care* and FIM change scores	.40(.34-.45)			Yes
*Domestic Life* and FIM change scores	.22(.16-.30)			Yes
**by Sex**	**Women n = 569**	**Men n = 321**		
*Self Care* and FIM change scores	.40(.32-.47)	.39(.28-.50)		Yes: Women and Men^c^,
*Domestic Life* and FIM change scores	.22(.13-.30)	.23(.13-.34)		Yes: Women and Men
**by Age**	**≤59 yrs**	**60 to 79 yrs**	**≥80 yrs**	
	**n = 118**	**n = 427**	**n = 346**	
*Self Care* and FIM change scores	.56(.41-.68)	.40(.31-.48)	-.32(.21-.43)	Yes: 60 to 79 yrs & ≥ 80 yrs
				No: ≤59yrs^d^
*Domestic Life* and FIM change scores	.44(.27-.58)	.23(.13-.33)	-.14(.02-.24)	Yes: 60 to 79 yrs & ≥ 80 yrs
				Yes: ≤59yrs^c^
**by Impairment**	**Orthopaedic n = 530**	**Neurological n = 176**	**Other Impairmentsn = 185**	
*Self Care* and FIM change scores	.31(.22-.39)	.48(.34-.60)	.42(.29-.53)	Yes: Orthopaedic.
				Yes: Neurological^c^ & Other Impairment^c^
*Domestic Life* and FIM change scores	.19(.10-.28)	.21(.06-.35)	.24(.09-.38)	Yes: all groups.

^a^Absolute magnitude of the negative correlation values are represented

^b^Using Cohen’s definition [44]: *r*_*s*_ = .10 to .29 (small); *r*_*s*_ = .30 to .49 (medium); *r*_*s*_ = .50 to 1.0 (large)

^c^Upper bound 95 % confidence interval suggests true value potentially lies above the range specified

^d^Lower bound 95 % confidence interval suggests true value potentially lies in the range specified

#### Hypothesis 10

Fifteen of 16 change score correlation values between the FIM and *Self Care* and *Domestic Life* scales by sex, age and major impairment groups were ≤ .49, as hypothesized (see Table 7). Participants aged ≤59 years had a value greater than .49 on the *Self Care* scale (*r*_*s*_ = .56), but the lower bound 95 % CI was lower than .49, (*r*_*s*_ = .41). Five upper bound 95 % confidence interval estimates were higher than .49: for *Self Care* (men, participants with neurological and other impairments); and for both *Self Care* and *Domestic Life* (participants aged ≤59 years).

#### Hypothesis 11

As hypothesized, there was a large effect size for both PC PART scales and FIM between admission and discharge with the FIM demonstrating the smallest effect size: *Self Care* scale (ES = 1.71; 95 % CI 1.60-1.82); *Domestic Life* scale (ES = 1.51; 95 % CI 1.40-1.61) and FIM (ES = 1.10; 95 % CI 1.01-1.20).

#### Hypothesis 12

Patients discharged home or to residential care had a large reduction (improvement) in mean PC-PART scores from admission to discharge on the *Self Care* scale (from 40.8 to 2.7; n = 810) and the *Domestic Life* scale (from 37.3 to 6.2; n = 814). These scores represented an average improvement from six *Self Care* participation restrictions at admission to none at discharge (ES = 1.73, 95 % CI 1.62-1.85), and from three *Domestic Life* participation restrictions at admission to none at discharge (ES = 1.56, 95 % CI 1.45-1.67). As hypothesized, both observed effect sizes were > 0.8.

#### Hypothesis 13

Patients discharged to acute hospital or transitional care had a large reduction in mean PC-PART scores from admission to discharge on the *Self Care* scale (from 52.4 to 18.4; n = 63) and the *Domestic Life* scale (from 53 to 27.5; n = 63). These scores represented an average reduction of nine *Self Care* participation restrictions at admission to two at discharge (ES = 1.54, 95 % CI 1.13-1.93), and from nine *Domestic Life* participation restrictions at admission to three at discharge (*ES* = 1.22, 95 % CI 0.83-1.59). Both effect sizes and their 95 % confidence intervalswere > 0.8. These results did not support the hypothesis of an effect size < .8 in this group.

### COSMIN summary

Overall, for both *Self Care* and *Domestic Life* PC-PART scales, the number of hypotheses supported were: 3 of 5 for construct validity; 3 of 3 for criterion validity; and 4 of 5 for responsiveness. Overall 6 of 8 hypotheses about validity and 4 of 5 hypotheses about responsiveness were supported. Sample sizes for all analyses were *good* to *excellent*.

## Discussion

This study evaluated construct validity, criterion validity and responsiveness of the PC-PART *Self Care* and *Domestic Life* scales for inpatient rehabilitation using the COSMIN framework. Overall, there was support for 10 of the 13 hypotheses.

Given that both the PC-PART and the FIM have provided evidence of reliability and validity, the lack of a strong negative correlation between the measures at admission could be interpreted as suggesting that the PC-PART measures a different construct to FIM. The FIM measures activity limitations [37]. The PC-PART scales performed in accordance with theoretical expectations, supporting construct validity of the PC-PART’s *Self Care* and *Domestic Life* scales as measures of ADL participation restriction.

To our knowledge, the PC-PART is the only instrument that specifically targets the transaction between people, their activity and the available supports in their living environments to record participation restrictions in activities of daily living required for community life. Other instruments seem similar, for example, the Assessment of Living Skills and Resources-Revised 2 (ALSAR-R2) [47]; Assessment of Life Habits (LIFE-H) [48]; Craig Handicap Assessment and Reporting Technique (CHART) [49]; and the Functional Autonomy Measurement System (SMAF) [50]. However, these assesments have applications in different areas of functioning than the PC-PART (e.g. performance in education, work, leisure tasks and body functions) and vary in the degree and manner in which they incorporate the need for, and availability of, supports, resources or assistance into their scoring [47–50]. The PC-PART therefore provides an important and unique contribution to health state measurement through its measurement of participation restrictions.

The relationship between comorbidity and PC-PART scores needs further investigation. Contrary to our expectations, the number and severity of comorbidities did not influence PC-PART scores (hypothesis 3). Overall, patients in this sample had relatively low comorbidity scores. Lack of variability in comorbidity scores may have affected the estimate of the correlation coefficient. It is possible that the Charlson Comorbidity Index was not sensitive to subclinical and chronic impairments that may impact people’s functioning (e.g. chronic pain or rheumatological conditions) [42]. Further evidence using prospective methods gathering more detailed information about comorbidity may add to our understanding about the measurement of participation restriction as related to the number and severity of co-existing impairments.

Admission PC-PART scores were shown to be higher for patients with stroke, compared to patients from other impairment groups, showing a lack of support for hypothesis 4, which postulated no difference between impairment groups. It may be that the sudden nature of stroke onset and combination of physical and cognitive impairments associated with stroke results in more participation restrictions in the accompishment of ADL than for people with other impairments. This result suggests that the PC-PART may be sensitive to impairment type, however this premise requires testing in a specifically designed study. If PC-PART scores are shown to differ between impairment groups, then it is possible the PC-PART may be useful for identifying groups of patients who are likely to require interventions focused on accomplishment of ADL required for community living as part of discharge planning.

The modest probability of both PC-PART scales’ scores ability to accurately reflect patients’ discharge destination shown in this study (hypothesis 6), seems likely to be an underestimation of their true discriminative ability. This result seems to have been influenced by the high proportion of missing PC-PART discharge data for patients discharged to acute inpatient care, as well as, a high proportion of patients with resolved participation restrictions in the transitional care group’s PC-PART discharge data [45]. The acute hospital and transitional care group discharge PC-PART scores were probably not representative of the group they were intended to represent, that is, patients transferred to acute care due to ongoing problems requiring medical management. On reflection, separation of patients discharged to acute hospital and transitional care into separate groups may have provided more robust validation data. Thus, these are preliminary findings. Prospective and specifically designed investigations of the PC-PART’s discriminative ability are required to produce more robust evidence about the ability of the *Self Care* and *Domestic Life* scale scores to accurately identify people who can return to community life from inpatient rehabilitation and those who continue to require inpatient services.

Both PC-PART scales appeared responsive. Their scores were shown to change in the direction expected when change had occurred, as indicated by other variables and instruments. Both scales demonstrated large effect sizes from rehabilitation admission to discharge. The correlation between change scores reflected a greater relative improvement in PC-PART scores than FIM scores between admission and discharge. This finding is consistent with theoretical expectations about PC-PART scores; that there should be a complete resolution of ADL participation restrictions prior to discharge home or to residential care (in residential care, the expectation is that all ADL needs are met). In contrast, it is possible for patients to be discharged to the community without complete independence scores on every FIM item, that is, without complete resolution of activity limitations, provided adequate supports are in place.

In this study, all patients’ *Self Care* and *Domestic Life* scores between admission and discharge showed large effect sizes, irrespective of discharge destination. For patients discharged to home or supported living environments, the large effect size of PC-PART scores between admission and discharge supported its responsiveness. It is possible that the patients discharged to transitional care (n = 69), who had no ADL participation restrictions at discharge because they were waiting for residential care placement, may have inflated the effect sizes for the acute and transitional inpatient care discharge group. Also, missing discharge PC-PART data for 13 of 17 patients transferred to acute care may have influenced the results by under-representing this group in the data. Thus, caution is advised when interpreting findings for hypothesis 13 due to limitations of the data as well as potential bias introduced during analyses. Responsiveness of the PC-PART scales should be further investigated in prospective, specifically designed studies.

One of the challenging decisions in validation research is whether to test hypotheses with the aid of structured guidelines, such as the COSMIN checklist, or whether to use more exploratory approaches. Formulating hypotheses prior to data analysis reduces the risk of bias when interpreting the results because criteria for validity are set before viewing the data. This avoids the temptation to think of alternative explanations for low correlations or no difference between groups, instead of concluding that the instrument may not be valid [27]. Limited existing validation research for the PC-PART influenced development of accurate hypotheses for this present study. The hypotheses used in this study were generated from some testing of the instrument [16, 32, 51–53], clinical knowledge and experience, combined with theoretical expectations of the instrument. The use of a more exploratory approach may have been useful for generating hypotheses for future testing, but would not have permitted the testing of evidence carried out in this study. Overall, the results of this study are positive, with the majority of hypotheses supported.

The COSMIN checklist provided a transparent, rigorous methodological structure for this research that assisted in minimizing methodological bias. It would be useful to use the COSMIN checklist to further evaluate the PC-PART scales in prospective, specifically designed research to build more evidence about the scales’ validity and responsiveness.

In clinical practice the PC-PART may aid discharge planning. The derived cut-off scores of zero *Self Care* and zero *Domestic Life* participation restrictions, desirable for discharge home or to residential care living situations, intuitively match clinical reasoning. The scales may be used to identify and prioritise areas for intervention and to ensure that patients who are discharged to community living environments do not have ADL participation restrictions at the time of discharge.

The PC-PART scales may be useful for clinical practice, clinical research and health care system management. In clinical practice, they may identify the presence of participation restrictions in ADL required for community life, enabling prioritisation of intervention and discharge planning. This may facilitate the reduction of barriers to discharge from inpatient care, which include issues of accommodation and supply of appropriate supports in community living environments [22]. In clinical research, changes that occur through interventions designed to reduce ADL participation restrictions, and their economic value, can be measured using the PC-PART scales. For the health care system, the PC-PART scales may be used to identify the nature and extent of participation restrictions experienced by populations in activities of daily living required for community life. This may aid understanding of the nature and extent of supports needed to enable people to live in the community and in turn, enable resources to be allocated where they are most needed.

### Limitations

The retrospective use of existing data limited the scope of analysis to the type and nature of the existing variables, which were collected for a different purpose. Use of existing data also meant that specific methodological requirements for some analyses (criterion validity) were not favorable. The combined grouping of patients discharged to acute and transitional care may have resulted in an underestimation of the discriminative ability and responsiveness of the PC-PART scales. Therefore, the results of this study need to be interpreted in light of its limitations. Prospective studies could ensure more detailed, useful and specific data for comparison with PC-PART scores are gathered. Finally, testing of PC-PART scores in relation to assessments such as the ALSAR-R2, SMAF or LIFE-H, which all focus on accomplishment of some ADL as well as broader life activities, may provide opportunity for further validation research.

## Conclusions

Overall, results of this rigorous validation study using the COSMIN checklist support the construct validity and criterion validity of the PC-PART’s *Self Care* and *Domestic Life* scales for inpatient rehabilitation and show they are responsive to clinical changes, as measures of ADL participation restrictions in activities of daily living required for community life. Evidence from this study adds to existing research establishing the PC-PART scales as unidimensional interval-level measures of participation restriction. Health service clinicians, managers and researchers may confidently use the PC-PART scales to measure the pattern and extent of people’s participation restrictions in activities of daily living required for community life, to gain an understanding of the burden of care associated with these needs and to aid resource allocation of services.

## Appendix A

**Table 8:** Example: two items from each of the PC-PART *Self Care* and *Domestic Life* scales

Item Label	Question to patient	Question to key informant	Observation^a^	Standard task (done with usual help)	Global response and score
*Self Care*					
Dressing top	Do you get your top dressed?	Does…get his/her top dressed?	Top adequately dressed?	Take off top and put it back on.	OK by self [0]
					OK with Help [0]
					Not OK [1]
Mobility (indoors)	Do you get around in your home OK?	Does…get around in the home OK?	N/A	Mobilise around objects in the room.	OK by self [0]
					OK with Help [0]
					Not OK [1]
*Domestic Life*					
Groceries	Do you get your groceries?	Does…get his/her groceries?	Adequate groceries present?	Clarify situation through discussion.	OK by self [0]
					OK with Help [0]
					Not OK [1]
Laundry	Do you get your clothes laundered regularly?	Does…get clothes laundered regularly?	Absence of dirty laundry?	Clarify situation through discussion.	OK by self [0]
					OK with Help [0]
					Not OK [1]

^a^When observations are not possible within a clinical setting, situation needs to be clarified through discussion

## Abbreviations

ADL: Activities of daily living
AUC: Area under the curve
CCI: Charlson comorbidity Index
COSMIN: COnsensus-based Standards for the selection of health Measurement INstruments
ES: Effect size
FIM: Functional Independence Measure
HLC: High level care
ICF: International classification of functioning, disability and health
LLC: Low level care
PC-PART: Personal Care Participation Assessment and Resource Tool
ROC: Receiver-operator characteristic curve
TCP: Transitional care program
